# Long-term cardiometabolic and bone health consequences of ketogenic diet in children with refractory epilepsy

**DOI:** 10.1186/s13052-025-02109-1

**Published:** 2025-08-22

**Authors:** Wael A. Bahbah, Ali M. El-Shafie, Heba M. S. El Zefzaf, Doaa M. Hosny, Shymaa A. Elshafey, Aya A. A. Hegazy

**Affiliations:** 1 Department of Pediatrics, Faculty of Medicine, Menoufia University, Yassin Abdel-Ghafar Street, Shebin El-Kom, Menoufia, 32511 Egypt; 2 Department of Radio Diagnosis and Interventional Radiology, Menoufia University, Menoufia, Egypt; 3 Department of Pediatrics, Ministry of Health and Population, Menoufia, Egypt

**Keywords:** Antiepileptic drugs, Bone health, Cardiometabolic, Consequences, Ketogenic diet, Refractory epilepsy

## Abstract

**Background:**

Ketogenic diet (KD) is a well-tolerated and efficacious therapy for refractory epilepsy (RE). While numerous mild short-term side effects have been reported, long-term cardiometabolic and bone heath consequences of KD need more advanced work-up and were not fully evaluated especially in children. So, we aimed to evaluate cardiac, vascular, metabolic, bone health and growth consequences in children with RE receiving KD for more than 2 years compared to those receiving antiepileptic drugs (AEDs ) without any dietary interference.

**Methodes:**

Fifty-six children following KD for at least 2 years, 27 classic KD and 29 modified atkins diet (MAD), were recruited in addition to 40 children with RE maintained on multiple AEDs. Lipid profile values, atherogenic indices, serum selenium binding protein 1, and anthropometric measurements were measured for all participants. Additionally, echocardiography, electrocardiography, carotid ultrasonography and DEXA scan were performed.

**Results:**

Atherogenic index of plasma (AIP) was high in all groups with no significant correlation with carotid intima-media thickness. Although no cardiac complications were documented, Bone mineral density (BMD) was significantly reduced in all groups. Castelli risk index II and ambulation were the significant predictors for reduced BMD in KD groups in contrast to AIP in AEDs group. Stunted growth was most prevalent in MAD group 44.8% while wasting was highest in AEDs group 40%.

**Conclusions:**

KD did not show additional risk regarding metabolic, cardiovascular, BMD and growth side effects compared to AEDs only. Therefore, KD remains a relatively safe dietary therapy for RE, yet close monitoring is still recommended.

**Supplementary Information:**

The online version contains supplementary material available at 10.1186/s13052-025-02109-1.

## Background

The ketogenic diet (KD), a high-fat, low-carb dietary therapy, is being increasingly accepted worldwide as a practical, effective and safe management alternative in children and adults with refractory epilepsy (RE), which is defined by the International League Against Epilepsy (ILAE) as ‘‘failure of adequate trials of two tolerated, appropriately chosen and used antiepileptic drug (AEDs) schedules to achieve sustained seizure freedom’’ [1, 2]. Through carbohydrate restriction, KD induces a ketotic state where cells rely on ketone bodies and fatty acids once glycogen stores have been depleted modulating the neurotransmission and providing anti-inflammatory neuroprotective effects with enhanced bioenergetic reserves [3].

There are four different ketogenic diets used for medical purposes according to the different amounts of macronutrients [4]. The classic KD is predominantly composed of long chain triglycerides with fat to carbohydrate ratio ranging from 3:1 to 4:1 [5]. As carbohydrate restriction is sometimes combined with additional calorie or protein restrictions, KD has undergone various modifications to improve tolerability and allow more dietary freedom with a ratio of 2:1 to 1:1 including the modified Atkins diet (MAD), the medium-chain triglyceride diet and the low glycemic index diet, which demonstrated comparable effectiveness in seizure control when compared to the classic KD [4, 6, 7].

In KD systematic reviews, more than 40 categories of adverse effects were identified with gastrointestinal manifestations, dehydration, electrolyte abnormalities, hypoglycemia, hyperuricemia, acidosis, and renal/genitourinary being the most common [8].

These Short-term side effects may occur at the start and after months of KD initiation and usually are mild and self-limited in most cases requiring only dietary modifications or pharmacological treatment [9]. Therefore, KD constitutes safe treatment for childhood RE.

Despite the safety of KD, it is not completely benign. As KD is not a balanced diet, the intake of vitamins and minerals can be compromised and vitamin D, selenium, magnesium, iron, and carnitine deficiency have been described [8, 9]. In addition, the effect of KD for an extended period on growth is quite controversial.

Furthermore, some rare and serious side effects were reported in the literature including pancreatitis, protein-losing enteropathy, prolonged QT interval, cardiomyopathy, increased risk of bone fractures, and changes in the basal ganglia, emphasizing the necessity of continuous clinical monitoring [2]. However, very little conflicting information is available about possible long-term side effects in children on the KD for longer than 2 years [10].

Therefore, this study aimed to assess cardiometabolic consequences in children with RE receiving prolonged KD whether classic KD or MAD longer than 2 years compared to children receiving multiple AEDs only who never received KD.

## Patients and methods

### Study design

This cross-sectional study was conducted from January 2023 to November 2023. The Menoufia Faculty of Medicine’s Institutional Review Boards (IRB) approved the study (ID number 12/2022 PEDI 4), and a written consent was obtained from parents of each participant prior to their involvement in the study. Our primary objective was assessment of long term metabolic, cardiac, and vascular consequences of KD as well as its impact on bone health and growth in children with RE. The secondary objective was to compare these consequences with RE children on multiple AEDs without any dietary interference.

### Study population

Fifty-six children aged 3 to 14 years attending the pediatric KD clinic at Menoufia University Hospital were enrolled. Recruitment criteria include children with RE receiving KD with 2–3 antiepileptic drugs (AEDs) for more than 2 years, 27 patients on classic KD (classic KD group) and 29 patients on MAD (MAD group). In addition to 40 age and sex-matching children with RE receiving multiple AEDs only without any dietary modifications (AEDs group). Valproate, levetiracetam, and carbamazepine were the most frequently utilized AEDs, their doses were administered in accordance with pediatric standard dosages and modified based on clinical response and drug serum level [11, 12]. Children with cardiovascular disease, bone disease, any systemic chronic condition other than epilepsy or receiving medications other than AEDs, except for vitamin D supplements, were excluded.

### Metabolic consequences assessment

Lipid profile was measured after 8–12 h fast using AU 680 Beckmann autoanalyzer (Beckmann), including total cholesterol (TC), triglycerides (TG), low density lipoprotein (LDL-C), and high-density lipoprotein (HDL-C). The mean level of lipid profile parameters throughout the 2 years (measured routinely every 6 months) was used in our study in all groups and results were interpretated as acceptable, borderline, high or low [13].

Then, atherogenic indices were calculated as follows, atherogenic index of plasma (AIP) = log (TG/HDL-C) with AIP value from − 0.3 to < 0.1 indicating low cardiovascular disease risk (CVD), 0.1 to < 0.24 indicating medium risk and ≥ 0.24 indicating high risk. Castelli risk index 1(CRI-1) was estimated as TC/HDLc ratio with low risk defined as CRI-1 < 3.515. Castelli risk index II(CRI-II) was determined as LDL-c/HDLc ratio with low risk defined as CRI-II < 3.015. Atherogenic coefficient (A.C) was calculated as (TC-HDLc)/HDLc with A.C < 3.015 defined as low risk [14].

### Cardiac consequences assessment

Electrocardiogram (ECG) was performed by (BT -08 SD ECG, UK) and the corrected QT interval (QTc) was calculated using Bazett’s formula: (QTc = QT interval / √RR interval). Normal QTc was defined as ≤ 0.44 Sect. [15]. Echocardiographic parameters including left Ventricular ejection fraction were measured in all patients by consultant pediatric cardiologist using two-dimensional, M-mode, colored Doppler, spectral Doppler and pulsed wave tissue Doppler imaging techniques (E4 LCD PRO, Tokyo, Japan) equipped with a high-frequency S8-3 transducer.

For Human Selenium-binding protein 1 (SELENBP1) assessment, three mL were withdrawn in plain tube and left to clot for 10–20 min at room temperature then centrifuge at 2000–3000 RPM for 20 min then the supernatant was collected without sediment and stored at -20 °C until used for measurement. Human SELENBP1 level was measured by Enzyme linked Immunosorbent Assay (ELISA) using ELISA Kit with catalog number E3250Hu supplied by Bioassay Technology Laboratory (BT LAB, Zhejiang, China).

### Vascular consequences assessment

External ultrasound was performed for the carotid artery intima-media thickness (CIMT) in all participants using a high-resolution ultrasound system (GE-LOGIQ E10 machine, USA) equipped with a 6–15 MHz probe. Normal CIMT in children ranged between 0.4 and 0.5 mm [16]. Distensibility, Carotid artery compliance (CAC) and arterial stiffness (SI) were calculated according to the following formulas: Distensibility = [(D_s_ - D_d_) / D_d_] x100, CAC = [(D_s_-D_d_) D_d_] / (P_s_- P_d_), and SI = ln (Ps/Pd)/[(Ds-Dd)/Dd] where Ds stands for systolic diameter, Dd diastolic diameter, Ps systolic blood pressure and Pd diastolic blood pressure [17].

### Bone mineral density (BMD) assessment

BMD was measured at the posterior-anterior lumbar spine (L1–L4) using dual- energy X-ray absorptiometry (DEXA) scans (Prodigy Series X-Ray Tube Housing Assembly, Mexico, S.A. de C.V. Spain). The results were interpreted using Z scores, where a Z-score of less than − 2 SD was considered abnormal. Osteopenia was defined as a Z-score between − 1 and − 2 SDs, while a normal BMD was defined as a Z-score above − 1 SD [18]. Serum 25(OH)D was quantitatively measured using a radioimmune assay (Roch Diagnostic Mannheim, Germany) and serum alkaline phosphatase (ALP), parathyroid hormone (PTH), phosphorus (PO4), calcium (Ca2+), arterial blood gases (ABG) were also assessed.

### Growth assessment

Anthropometric measurements including weight, height and body mass index (BMI) were documented and interpreted based on Egyptian Z score growth references [19, 20].

### Statistical analysis

The collected data was tabulated and analyzed using IBM SPSS software package version 20.0. **(**Armonk, NY: IBM Corp**).** The Chi-square test was used for categorical variables, to compare between different groups and when more than 20% of the cells have expected count less than 5 monte carlo correction was used. For normally distributed quantitative variables, student t-test was used to compare between two groups, F-test (ANOVA) to compare between more than two groups, and Post Hoc test (Tukey) for pairwise comparisons. For abnormally distributed quantitative variables, the Mann Whitney test was used to compare between two groups, Kruskal Wallis test to compare between more than two groups and Post Hoc (Dunn’s multiple comparisons test) for pairwise comparisons. The significance of results was judged at the 5% level. The spearman’s coefficient was used to determine the correlation. Linear regression analysis was done to detect the most independent factor affecting each consequence.

Using statistics and sample size program version 6 and based on a previous study [21], the least sample size was 36 subjects divided into two equal groups with the power of study 80% and confidence level 95%.

## Results

**Fig. 1:**
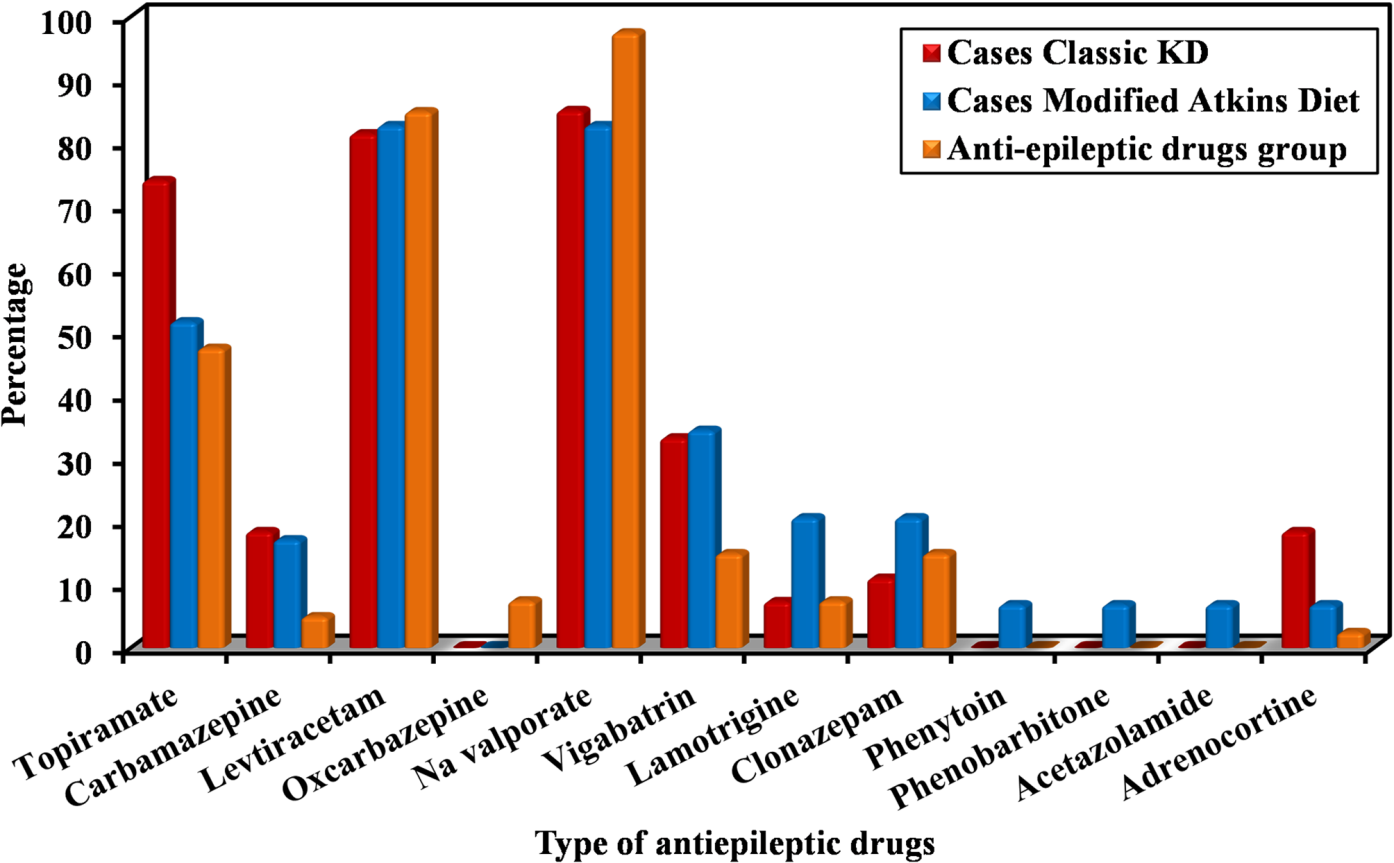
Comparison between the three studied groups according to type of antiepileptic drugs. This figure showed that the most type of antiepileptic drugs used was Na Valproate in 86 patient (23 in Classic KD group, 24 in MAD group and 39 in AED group ) followed by Levetiracetam in 80 patient (22 in Classic KD group, 24 in MAD group and 34 in AED group) then Topiramate in 54 patient (20 in Classic KD group, 15 in MAD group and 19 in AED group)

Ninety-six children with RE were enrolled in our study, the median (IQR) age was 5. 0 (4.0–9.0) years in KD groups and 6.0 (3.0–9.0) years in AEDs group with no significance difference between the 3 studied groups regarding age or sex. The median (IQR) number of AEDs used was 2.0 (2.0–4.0) in KD groups versus 4.0 (3.0–5.0) in AEDs group and Na Valproate was the most common drug used (Fig. 1**).**

### KD and metabolic consequences

Lipid profile showed significant differences in triglyceride and HDL-C levels among studied groups. However, the median triglyceride and LDL-C levels throughout the 24 months were within the acceptable range for age in all groups. Unlikely, MAD group showed borderline mean cholesterol and median HDL-C levels, also AEDs group showed borderline median HDL-C level, while all lipid parameters were within the acceptable levels in classic KD group.

Despite neither of lipid profile parameters was high for age in all groups, atherogenic indices showed a significant elevation as CRI-I was high 3.57 (2.02–3.75) and 3.56 (2.61–3.86) in MAD and AEDs groups respectively. Also, AIP was significantly high in classic KD, MAD and AEDs groups with median 0.26,0.23 and 0.30 respectively indicating moderate/high cardiovascular risk (Table 1).

**Table 1:** Lipid profile and atherogenic indices (metabolic consequences) among the three studied groups

	Classic KD (*n* = 27)	Modified Atkins Diet (*n* = 29)	Anti-epileptic drugs group (*n* = 40)	*p* value
**Cholesterol )mg/dl)** Mean ± SD.	169.2 ± 76.82	175.9 ± 51.14	159.1 ± 28.77	0.414
**Triglyceride (mg/dl)** Median (IQR)	95.0 (80.0–163.0)	95.0 (60.0–118.0)	90.0 (80.0–106.0)	**0.003** ^*^
**Significance between Groups**	p_1_ = 0.341, p_2_ = 0.034^*^, p_3_ < 0.001^*^			
**HDL-C** (**mg/dl)** Median (IQR)	55.0 (48.0–59.0)	40.0(37.0 – 99.0)	45.0(40.50–56.50)	**0.003** ^*^
**Significance between Groups**	p_1_ = 0.001^*^, p_2_ = 0.515, p_3_ = 0.006^*^			
**LDL-C (mg/dl)** Median (IQR)	73.0 (22.0–93.0)	80.0 (47.0–99.0)	77.0 (32.0–90.50)	0.211
**Castelli risk I(CRI-I)** Median (IQR)	2.28 (1.70–4.86)	3.57 (2.02–3.75)	3.56 (2.61–3.86)	0.806
**Castelli risk index II (CRI-II)** Median (IQR)	1.25 (0.54–1.97)	1.46 (0.64–2.17)	1.44 (0.89–2.06)	0.557
**Atherogenic index of plasma (AIP)** Median (IQR)	0.26 (0.04–0.53)	0.23 (0.19–0.35)	0.30 (0.20–0.39)	0.231
**Atherogenic coefficient (AC)** Median (IQR)	1.20 (0.70–3.86)	2.56 (1.02–2.75)	2.74 (1.62–2.97)	0.483

KD: ketogenic diet HDL-C: High density lipoprotein cholesterol LDL-C: Low density lipoprotein cholesterol

IQR: Inter quartile range SD: Standard deviation

p: p value for comparing between the three studied groups

p_1_: p value for comparing between Classic KD and Modified Atkins

p_2_: p value for comparing between Classic KD and Anti-epileptic drugs group

p_3_: p value for comparing between Modified Atkins and Anti-epileptic drugs group

*: Statistically significant at *p* ≤ 0.05

The normal ranges are: Serum cholesterol (< 170 mg/dl is acceptable, 170–199 mg/dl is borderline and > 200 mg/dl is high), serum triglyceride (in patients 9 years or younger: <75 mg/dl is acceptable, 75–99 mg/dl is border line and > 100 mg/dl is high; in patients 10 years or older: <90 mg/dl is acceptable, 90–129 mg/dl is border line and > 130 mg/dl is high), serum LDL(< 110 mg/dl is acceptable, 110 – 129 mg/dl is borderline and > 130 mg/dl is high), serum HDL (> 45 mg/dl is acceptable, 40–45 mg/dl is 39–40 borderline and < 40 mg/dl is low)

### KD and cardiac consequences

**Fig. 2:**
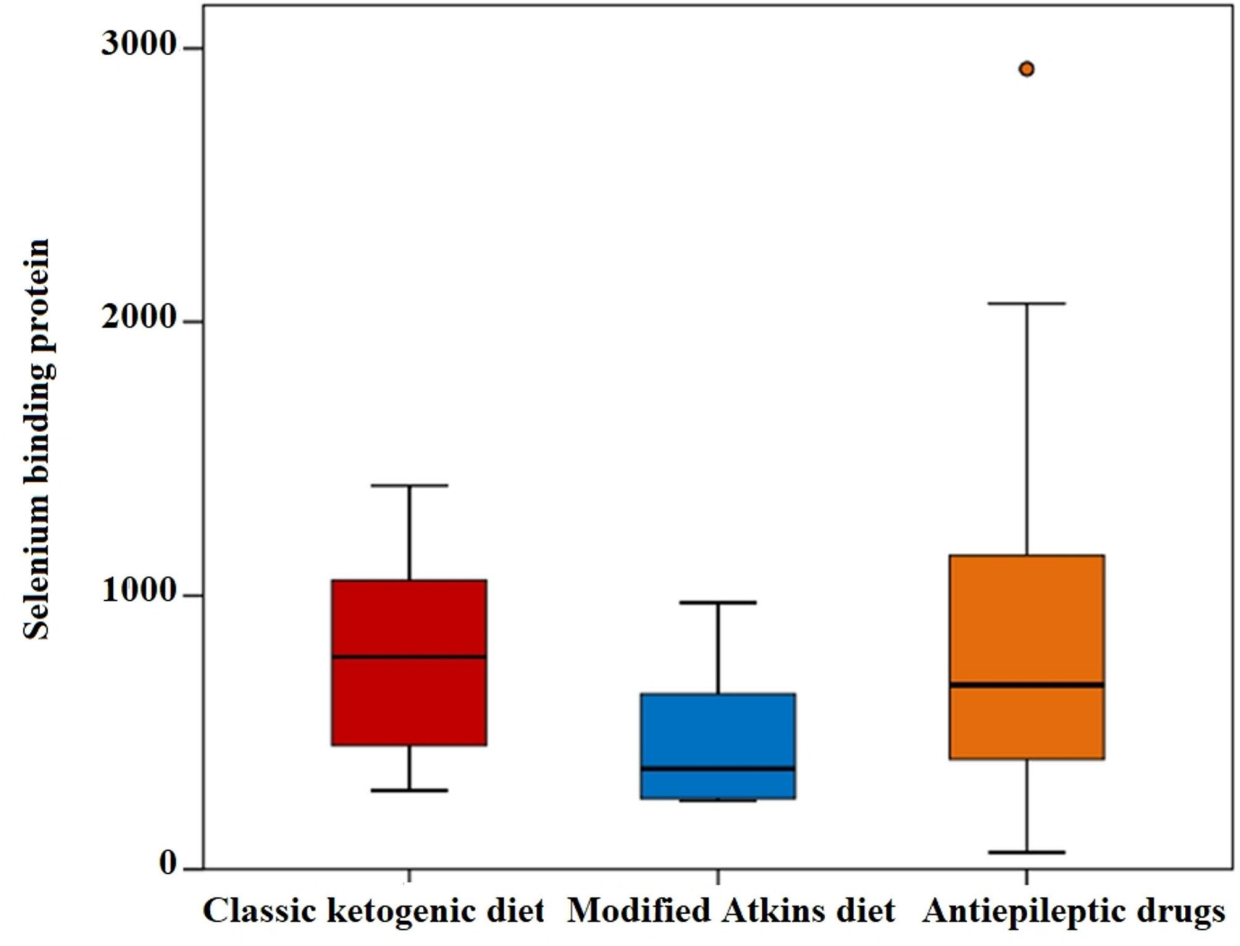
Comparison between the three groups studied according to selenium binding protein 1 level

**Fig. 3:**
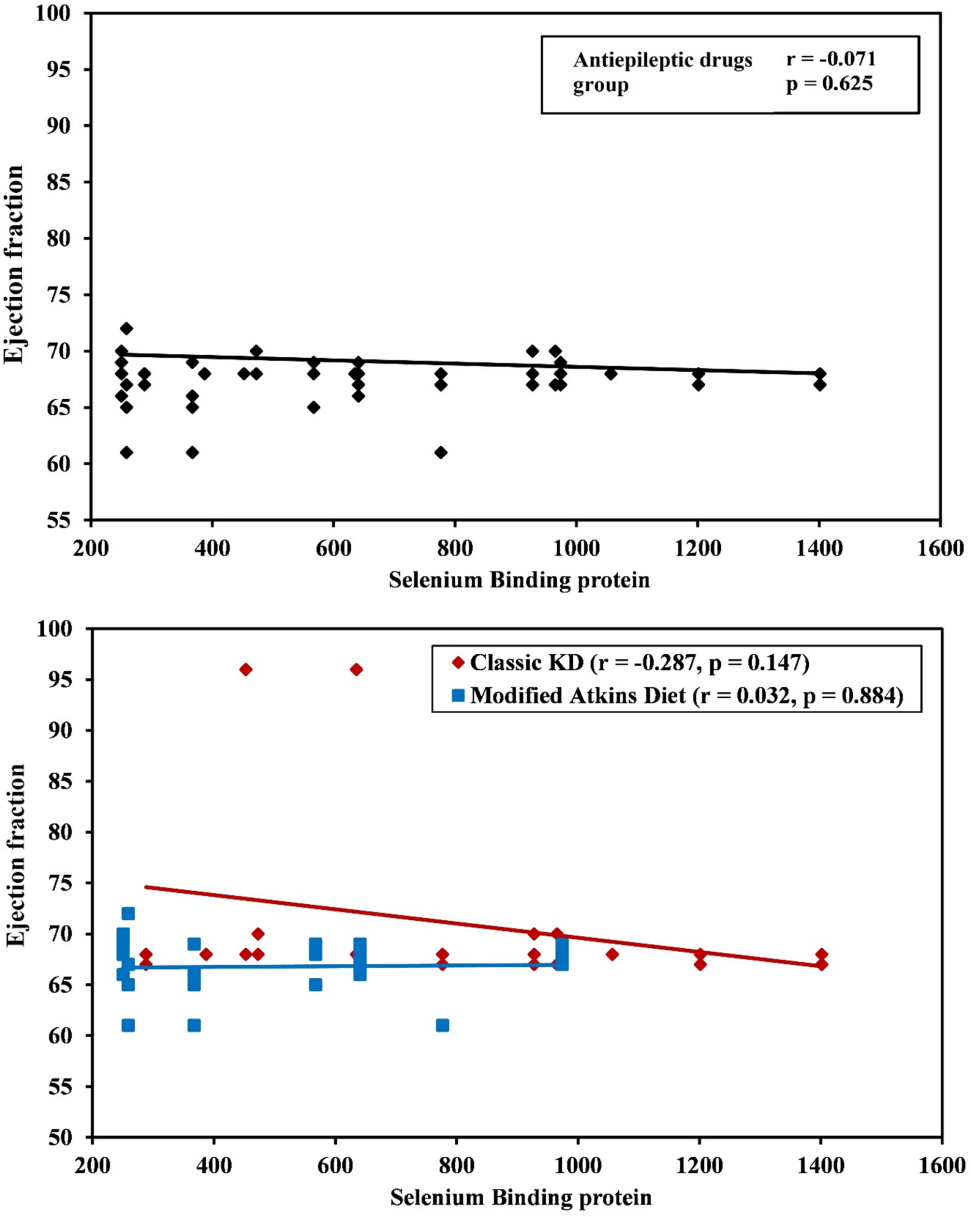
Correlation between ejection fraction by echocardiography and selenium binding protein 1 in AED patients’ group, Classic KD group and modified Atkins diet group

No cardiomyopathy or prolonged QTc interval were reported, and ejection fraction by echocardiography (despite the significance between 3 groups) was normal in all study groups. The median level of SELENBP1 was significantly lower in MAD group 367.3 (258.6–640.9) ng/l) compared to classic KD and AED groups (*p* = 0.005) but no significant correlation was found between ejection fraction by echocardiography and SELENBP1 in the 3 groups (*p* = 0.147, *p* = 0.884 and *p* = 0.625 in classic KD, MAD and AEDs groups respectively) (Table 2) (Figs. 2 and 3).

**Table 2:** Cardiovascular assessment among the three studied groups

	Classic KD (*n* = 27)	Modified Atkins Diet (*n* = 29)	Anti-epileptic drugs group (*n* = 40)	*p* value
**QTc interval (ms)** Mean ± SD.	0.40 ± 0.05	0.42 ± 0.05	0.42 ± 0.04	0.266
**Ejection fraction by echocardiogram (EF %)** Mean ± SD.	71.07 ± 9.02	66.78 ± 2.86	68.89 ± 2.88	0.028*
**Significance between groups**	p1 = 0.021*, p2 = 0.272, p3 = 0.330			
**Selenium binding protein 1 (ng/l)** Median (IQR)	776.6(452.4–1056.7)	367.3(258.6–640.9)	673.8(402.5–1147.0)	**0.005** ^*^
**Significance between groups**	p1 = 0.003*, p2 = 0.591, p3 = 0.007*			
**Average of carotid intima media thickness (CIMT) (mm)** Mean ± SD.	0.49 ± 0.01	0.49 ± 0.02	0.49 ± 0.02	0.251
**Carotid artery compliance (CAC) (mm**^**2**^**/mmHg)** Median (IQR)	0.06 (0.05–0.06)	0.05 (0.03–0.06)	0.05 (0.03–0.06)	0.042*
**Significance between groups**	p1 = 0.025*, p2 = 0.028*, p3 = 0.837			
**Distensibility** Median (IQR)	6.89 (6.89–8.33)	5.26 (3.57–7.10)	5.26 (3.57 − 7.0)	0.065
**Carotid artery stiffness (SI)** Median (IQR)	6.90 (6.65–7.93)	9.50 (6.65–12.49)	9.50 (6.77–12.49)	0.132

KD: ketogenic diet IQR: Inter quartile range SD: Standard deviation

QTc interval: corrected QT interval

p: p value for comparing between the three studied groups

p1: p value for comparing between Classic KD and Modified Atkins

p2: p value for comparing between Classic KD and Anti-epileptic drugs group

p3: p value for comparing between Modified Atkins and Anti-epileptic drugs group

*: Statistically significant at *p* ≤ 0.05

### KD and vascular consequences

**Fig. 4:**
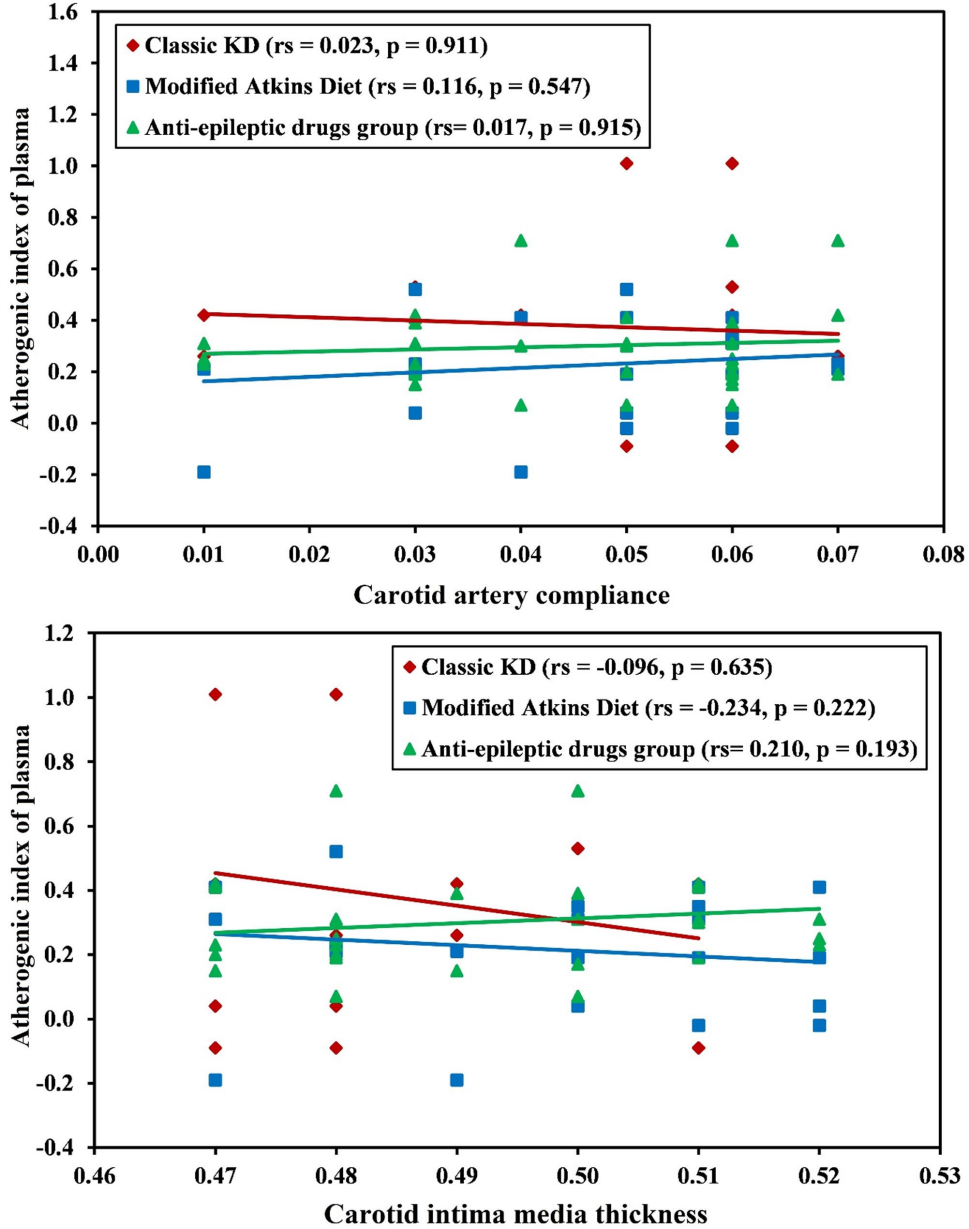
Correlation between atherogenic index of plasma and carotid artery compliance and carotid intima media thickness in each group

The mean CIMT was nearly the same among the 3 groups 0.49 mm which is considered normal for age. Additionally, no significant difference was found between the 3 study groups regarding distensibility or carotid stiffness. However, Patients in classic KD group showed significant difference regarding CAC (*p* = 0.042) (Table 2). Correlation between CAC and lipid profile parameters revealed a positive correlation with LDL-C in MAD group (*p* = 0.033) while with Cholesterol in AEDs group (*p* = 0.004). On the other hand, despite the high AIP, no correlation exists with CAC or CIMT among the 3 groups (Table 3) (Fig. 4).

**Table 3:** Correlation between bone mineral density (BMD) and carotid artery compliance (CAC) with different parameters in each group

	Classic Ketogenic Diet group			Modified Atkins Diet group		AEDs group	
	*r*_s_	*p*		*r*_s_	*P*	*r*_s_	*p*
**Bone mineral density**							
**Body mass index (BMI) (kg/m** ^**2**^ **)**	-0.635^*^	**< 0.001** ^*^		0.346	0.066	-0.011	0.937
**Ambulation**	-0.343	0.059		0.092	0.694	0.267^*^	**0.045** ^*^
**Parathyroid hormone**	-0.438^*^	**0.022** ^*^		0.557^*^	**0.002** ^*^	0.122	0.372
**Cholesterol**	-0.850^*^	**< 0.001** ^*^		0.154	0.426	-0.369^*^	**0.005** ^*^
**Triglyceride**	-0.452	**0.018** ^*^		-0.094	0.628	0.107	**0.434**
**High density lipoprotein cholesterol (HDL-C)**	0.620^*^	**0.001** ^*^		0.378^*^	**0.043** ^*^	0.347^*^	**0.009** ^*^
**low density lipoprotein cholesterol** (**LDL-C)**	-0.366	0.061		-0.522^*^	**0.004** ^*^	-0.164	0.227
**Castelli risk index II (CRI-II)**	-0.689^*^	**< 0.001** ^*^		0.307	0.105	-0.075	0.580
**Atherogenic index of plasma**	-0.446^*^	**0.020** ^*^		-0.098	0.612	-0.275^*^	**0.040** ^*^
**PH**	0.520^*^	**0.005** ^*^		0.088	0.650	0.334^*^	**0.012** ^*^
**Carotid artery compliance (CAC)**							
**Cholesterol**	0.177		0.192	0.269	0.159	0.541^*^	**0.004** ^*^
**low density lipoprotein cholesterol** (**LDL-C)**	0.089		0.657	0.398^*^	**0.033** ^*^	0.157	0.249
**PCO**_**2**_	-0.506^*^		**0.007** ^*^	-0.094	0.628	-0.212	0.117

AEDs : antiepileptic drugs PCO_2_: partial pressure of carbon dioxide

PH: potential hydrogen

r_s_: Spearman coefficient

*: Statistically significant at *p* ≤ 0.05

### KD and BMD

Our study revealed a significance regarding serum vitamin D, calcium and alkaline phosphatase levels among the 3 studied groups, however all levels were within normal range for age. Also, no significance was observed regarding ambulation status. The mean bicarbonate (HCO3) level was significantly lower in MAD group compared to other 2 groups (*p* = 0.001). (Table 4).

**Table 4:** Bone health assessment among the three studied groups

	Classic KD (*n* = 27)			Modified Atkins Diet (*n* = 29)		Anti-epileptic drugs group (*n* = 40)		*p*
**Calcium (mg/dl)** Mean ± SD.	9.47 ± 0.68			9.44 ± 0.81		9.0 ± 0.76		**0.019** ^*^
**Significance between groups**	p_1_ = 0.994, p_2_ = 0.042^*^, p_3_ = 0.049^*^							
**Phosphorus (mg/dl)** Mean ± SD.	5.20 ± 0.88			4.86 ± 0.84		5.01 ± 0.64		0.259
**Alkaline phosphatase (IU/L)** Mean ± SD	190.5 ± 46.89			153.6 ± 28.90		162.4 ± 39.77		**0.002** ^*^
**Significance between groups**	p_1_ = 0.002^*^, p_2_ = 0.013^*^, p_3_ = 0.628							
**Vitamin D** (**ng/ml)** Median (IQR)	50.0 (40.70–61.0)			34.0 (28.0–50.20)		66.0 (45.0–79.0)		**> 0.001** ^*^
**Significance between groups**	p_1_ = 0.001^*^, p_2_ = 0.247, p_3_ > 0.001^*^							
**Parathyroid hormone (pg/ml)** Median (IQR)	24.30 (19.0–33.0)			24.0 (18.0–35.0)		27.3 (19.35–38.1)		0.741
**PH** Mean ± SD.	7.40 ± 0.02			7.38 ± 0.05		7.39 ± 0.04		0.292
**PCO**_**2**_**(mmHg)** Mean ± SD.	38.20 ± 1.98			41.69 ± 9.13		38.36 ± 5.05		**0.006** ^*^
**Significance between groups**	p_1_ = 0.086, p_2_ = 0.994, p_3_ = 0.068							
**HCO**_**3**_**(mEq/L)** Mean ± SD.	22.26 ± 2.23			19.86 ± 4.25		22.56 ± 2.28		**0.001** ^*^
**Significance between groups**	p_1_ = 0.001^*^, p_2_ = 0.916, p_3_ = 0.001^*^							
**Ambulation** No	10	37.0	6		20.7	11	27.5	0.394
Yes	17	63.0	23		79.3	29	72.5	
**Bone mineral density (z score)** Median (IQR)	-3.70 (-3.90 – -3.40)			-3.70 (-3.90 – -3.40)		-3.10 (-3.70 – -2.90)		**0.021** ^*^
**Significance between groups**	p1 = 0.862, p2 = 0.016*,p3 = 0.024*							

KD: ketogenic diet IQR: Inter quartile range SD: Standard deviation

PCO_2_: partial pressure of carbon dioxide HCO_3 :_ bicarbonate

p: p value for comparing between the three studied groups

p_1_: p value for comparing between Classic KD and Modified Atkins

p_2_: p value for comparing between Classic KD and Anti-epileptic drugs group

p_3_: p value for comparing between Modified Atkins and Anti-epileptic drugs group

*: Statistically significant at *p* ≤ 0.05

BMD was significantly impaired in the 3 groups with median (IQR) z score for age − 3.70 (-3.90 – -3.40), -3.70 (-3.90 – -3.40) and − 3.10 (-3.70 – -2.90) in classic KD, MAD and AEDs groups respectively (Table 4). BMD showed positive correlation with multiple parameters in all groups (Table 3). So, univariate regression analysis was done revealing a significance with ambulation status and increased BMI, AIP and CRI-II in both KD groups while in AEDs group, significance was evident with increased BMI and AIP. Multivariate regression analysis identified ambulation and CRI-II as predictors for decreased BMD in KD groups while AIP was the only predictor for decreased BMD in AEDs group (Table 5).

**Table 5:** Univariate and multivariate linear regression analysis for the parameters affecting bone mineral density among the studied groups

	Univariate		^#^Multivariate	
	*p*	B (LL – UL 95%C.I)	*p*	B (LL – UL 95%C.I)
**Ketogenic Diet groups**				
**Body mass index (BMI) (kg/m2)**	**< 0.001** ^*^	-0.146(-0.173 – -0.119)	0.308	-0.016(-0.049–0.016)
**Atherogenic index of plasma**	**0.002** ^*^	0.027(0.010–0.043)	0.069	0.018(-0.002–0.038)
**Castelli risk index II (CRI-II)**	**< 0.001** ^*^	-0.437(-0.545 – -0.329)	**< 0.001** ^*^	-0.384(-0.552 – -0.216)
**Ambulation**	**0.001** ^*^	0.676(0.302–1.051)	**0.006** ^*^	-0.343(-0.579 – -0.108)
**AEDs group**				
**Body mass index (BMI) (kg/m2)**	**0.005** ^*^	0.118(0.039–0.197)	0.167	0.065(-0.029–0.160)
**AIP**	**< 0.001** ^*^	-0.041(-0.050 – -0.031)	**< 0.001** ^*^	-0.023(-0.027 – -0.019)
**Castelli risk index II (CRI-II)**	0.151	0.176(-0.068–0.420)		
**Ambulation**	0.177	0.426(-0.205–1.057)		

B: Unstandardized Coefficients

C.I: Confidence interval LL: Lower limit UL: Upper Limit

#: All variables with *p* < 0.05 were included in the multivariate *: Statistically significant at *p* ≤ 0.05

### KD and growth

Growth revealed a high significant change among the 3 studied groups, wasting was significantly higher in AEDs (40%) than KD groups, while obesity was most prevalent in classic KD group (18.5%) than the 2 other groups (*p* = 0.002). Regarding height, stunting was significantly higher in MAD group (44.5%) than other 2 groups (*p* < 0.001) (Table 6).

**Table 6:** Growth assessment among the three studied groups

	Classic Ketogenic Diet group (*n* = 27)		Modified Atkins Diet group (*n* = 29)		Anti-epileptic drugs group (*n* = 40)		*p*
	No.	%	No.	%	No.	%	
**Height for age Z Score**							
Normal	22	81.5	16	55.2	37	92.5	^MC^p **<0.001** ^*^
Stunted	5	18.5	13	44.8	3	7.5	
**Height (cm)** Mean ± SD.	87.15 ± 7.45		112.4 ± 19.26		113.0 ± 19.04		**< 0.001** ^*^
**Significance between groups**	p_1_ < 0.001^*^, p_2_ < 0.001^*^, p_3_ = 0.989						
**Body mass index (BMI) Z Score**							
Normal	17	63.0	16	55.2	21	52.5	^MC^p = 0.002^*^
Wasted	5	18.5	11	37.9	16	40.0	
Overweight and obese	5	18.5	2	6.9	3	7.5	
**Body mass index (BMI) (kg/m**^**2**^**)** Mean ± SD.	16.89 ± 3.48		14.39 ± 2.93		14.50 ± 3.64		**0.009** ^*^
**Significance between groups**	p_1_ = 0.020^*^, p_2_ = 0.016^*^, p_3_ = 0.991						

Stunted means height for age z score < -2 SD wasted means BMI for age z score < -2 SD

IQR: Inter quartile range SD: Standard deviation MC: Monte Carlo

p: p value for comparing between the three studied groups

p_1_: p value for comparing between Classic KD and Modified Atkins

p_2_: p value for comparing between Classic KD and Anti-epileptic drugs group

p_3_: p value for comparing between Modified Atkins and Anti-epileptic drugs group

*: Statistically significant at *p* ≤ 0.05

## Discussion

KDs are widely used as a successful treatment for RE in children. Despite its demonstrated effectiveness, it is not free of side effects [22]. Since dyslipidemia is a known risk factor for cardiovascular diseases (CVD) in the general population, cardiovascular adverse effects particularly are expected in children who follow this high-fat diet for an extended period [4, 23]. However, the long-term impacts of KD on vascular atherogenic risk and metabolic processes are little understood [22]. As a result, it’s crucial to carefully evaluate the possible adverse effects of KD as well as possible mitigation strategies. So, we evaluated cardiovascular, metabolic, bone health and growth consequences in children on prolonged KD.

Theoretically, KD was linked to hyperlipidemia with an estimated up to 60% of children reported to have variations in their lipid profiles, especially during the first 12 months of the diet [8, 24]. Also, some studies reported a notable increase in total cholesterol, LDL, Very low-density lipoprotein (VLDL), triglycerides, and total apoB lipoproteins six months after beginning KD [25, 26] Nonetheless, research over longer time periods revealed that despite these frequent lipid profile changes, there was no significant difference of the median lipid profile levels in classic KD and MAD at 24 months while maintained within the acceptable range [24, 27–29]. On the other hand, Coppola et al. reported hyperlipidemia as a side effect in patients receiving prolonged liquid ketogenic formula for RE [30].

This controversy may be explained by the type of fat intake and changes in lipid profiles in KD, as patients who consumed a lot of olive oil and a small amount of saturated fat had significantly lower lipid profiles 24 months after the start of KD, even though some of the patients had pre-existing dyslipidemia [31, 32]. Nevertheless, the impact of fat sources on lipid profile levels remains unclear [33]. Mean 24 months lipid profile changes were prevalent in our study not only in both KD groups but also in AEDs group with the highest levels of cholesterol, triglyceride and LDL-C documented in the MAD group which could be attributed to the type of fat these patients were consuming, or due to the additional effect of AEDs co-administration.

Some AEDs may negatively alter lipid profile levels in patients with epilepsy [34]. Although the relationship between AEDs and hyperlipidemia in children has not been thoroughly investigated, research on adults has shown that patients treated with enzyme-inducing anticonvulsants had a higher likelihood of being subsequently diagnosed with hyperlipidemia [35]. Also, valproic acid was linked to significant alterations in plasma lipid levels in epileptic patients [34] which was among the commonest used drugs in our study.

Recently, it has been proposed that a standard lipid profile may not be adequate to investigate the relationship between KD and CVD. As a result, numerous studies attempted to improve the markers of atherogenic dyslipidemia through computed lipid ratios (atherogenic indices), which are a more reliable indicators of atherosclerosis onset than any single lipid measurement [14, 36]. Additionally, the AIP, which represents the actual relationship between atherogenic and protective lipoproteins, was referred to as a potent predictor of the risk of coronary heart disease and atherosclerosis [37].

Our results revealed that AIP was higher in all studied groups with significant elevation in AEDs group than KD groups, indicating a moderate to increased CVD risk in children with RE receiving either KD or AEDs.

Carotid atherosclerosis (CA), which can be evaluated noninvasively with carotid ultrasonography, was identified as a reflector of generalized atherosclerosis [38]. Older generation AEDs and AEDs polytherapy, through raising plasma homocysteine levels, were linked to an increase in carotid intima media thickness, which raises the risk of atherosclerosis at a young age compared to new generation AEDs monotherapy [39].

However, studies for atherogenic vascular risk in children receiving KD are scarce [22]. At three and twelve months following the start of KD, Kapetanakis et al. reported increased CIMT and SI with worsened lipid profiles however, after two years of treatment these changes recovered to baseline [22]. Contrary, Ozedmir study found that KD based on olive oil did not seem to alter the elasticity of the aorta and carotid arteries in children with epilepsy, while KD was still linked to a higher level of serum lipids [21].

In our study, no changes were observed in CIMT, carotid artery distensibility or SI in the 3 studied groups. However, CAC, which is a local measure of stiffness by direct measurement of carotid arterial wall stiffness [22], was significantly affected in the classic KD group than other groups. Also, AIP showed no significant correlation (despite its elevated level) with CIMT or CAC among the 3 groups studied. To our knowledge the correlation between the AIP and carotid atherosclerosis wasn’t previously studied in patients with RE on KD or on AEDs. Therefore, monitoring of lipid levels and carotid measures in children with RE is recommended.

Although KD-induced cardiac complications are rare, emerging evidence claims that a starvation-like state linked to KD may cause metabolic disruption that might lead to abnormal cardiac conduction and/or myocardial damage [40, 41]. QT interval prolongation is particularly concerning due to the elevated risk of cardiac dysrhythmia and abrupt mortality [40]. According to Best et al. study, three out of twenty-one children with KD had a prolonged QT interval, with two of them had normal pre-diet ECGs which showed that QT had developed after the diet was started. They also had left atrial and left ventricular enlargement and one of them returned to normal after the diet was stopped [40].

Despite being alarming, this finding was not published in another study. A later study including 80 patients did not show any change in QT interval from their baseline after at least one year on a KD [41, 42]. In addition, none of the patients in our study experienced prolonged QT interval or any ECG abnormality 24 months after KD.

KD is deficient in certain minerals, vitamins, and trace elements, one of them being selenium which is an essential cofactor for antioxidant enzyme glutathione peroxidase, which eliminates free radicals from the body to prevent oxidative tissue damage. Consequently, reactive free radicals, which are harmful to cardiac myocytes, would increase because of selenium deficiency [43]. Selenium deficiency-induced cardiac complications, particularly cardiomyopathy, have been reported in children on KD while KD associated cardiac complications in the absence of selenium deficiency, have not been reported [40, 44].

However, controversial data is available regarding the effects of the KD on cardiac functions [40]. Although selenium levels were significantly lower six and twelve months after KD treatment compared to pre-treatment levels [45], another study found no significant difference between baseline measurements and cardiac outcomes, such as serum carnitine, serum selenium, electrocardiography, and echocardiographic exams, one year after KD [42].

This disparate selenium deficiency ratios between the studies may be owing to the different geographical regions (Because the dietary selenium content is dependent on the soil selenium content), the different threshold values for selenium deficiency, varying KD contents, and lastly, different KD ratios.

No relevant echocardiographic findings were observed in our patients. However, SELENBP1 level was significantly lower in the MAD group than classic KD group. This can be explained as patients on classic KD in our study depend mainly on KD special formula (KetoCal) which meet the current Recommended Dietary Allowance (RDA) requirements for selenium [46]. Thus, the selenium levels should be closely monitored in patients on KD as well as supplementation of this trace mineral for deficient children.

Vitamin D supplementation was suggested to improve vitamin D status while on KD as KD may predispose to osteopenia due to low calcium and vitamin D levels, which puts them at risk for pathological fractures [3, 47, 48]. Unfortunately, despite the bone-sparing benefits of improved vitamin D status, some evidence links KD usage to growth failure, changes in body composition, and osteopenia in some patients [49].

The 2009 International Ketogenic Diet Study group consensus-based guidelines suggested that it could be beneficial for children to have periodic DEXA screening for assessment of bone health, despite this only one study involving three adults has been published denoting no abnormality in their BMD at baseline and over the course of five years of the KD [50]. In fact, adult studies are unable to shed light on skeletal growth because the majority of patients following the ketogenic diet are children, who have far higher rates of skeletal turnover and the ultimate goal of bony mass accumulation.

Later, Bergqvist and colleagues examined the bone health of 25 prepubertal children with RE 15 months after starting KD, they found that nearly half of the individuals had poor baseline BMD for age, and that BMD was predicted by age, body mass index Z scores, and ambulation status [47]. This conclusion was then supported by a study by Simm et al. that showed that for each year the children were on the KD, their BMD decreased by an average of 0.16 SD (compared to age-matched referent children) [48].

Chronic acidosis, which depletes bone minerals for buffering capacity, was thought to be the cause of KD’s detrimental effects on bone health. Additionally, acidosis hampered the conversion of serum 25-OHD to 1,25-dihydroxyvitamin D [47]. Similar findings were observed in our study as a significant impairment of BMD in classic KD, MAD and AEDs groups despite normal levels of both vitamin D and calcium in all studied groups while a positive correlation with acidosis exists.

AEDs negative impact on bone health was documented in both adults and children which was explained either by direct affection of the cells involved in bone production and absorption or indirectly by impaired metabolism of calcium and vitamin D [47]. Additionally, epileptic adults who have received AEDs since childhood have less bone mass compared to those who started treatment in adulthood which may indicate a particular effect of AEDs on the developing skeleton as well as a potential cumulative effect of AED use on the skeleton [51]. Although the newer AEDs have better side effect profiles, cross-sectional data for some of the newer AEDs indicate that they also may increase the risk of poor bone health, but longitudinal data are lacking and their effects on bone health in growing children have not been well established [52]. So, low BMD in our study may be in a part due to concurrent use of AEDs or presented from baseline before starting KD.

As atherosclerosis and osteoporosis share common pathogenetic pathways implicated in bone and vascular mineralization, the impact of high fat content of KD on bone health is also questionable. This detrimental effect may be mediated directly through the increased oxidative stress and systemic inflammation that dyslipidemia is associated with, leading to increased osteoclastic activity and reduced bone formation, or through the atherosclerotic process, which affects bone’s vascularization [53].

However, the role of dyslipidemias in bone health is less documented, with many studies yielding inconsistent results. Despite the high heterogeneity and the variable quality of evidence, dyslipidemia, mainly high TC and LDL-C and, to a lesser extent, TG concentrations seem to be associated with low bone mass and increased fracture risk [53]. Also, a negative correlation between the AIP and total BMD was documented in adult women [54] and AIP values > 0.11 were independently related to a degraded bone microarchitecture as measured by trabecular bone score suggesting that AIP might be a useful tool in the overall assessment of bone metabolism in postmenopausal women [55].

Our results revealed a significant correlation between high cholesterol, LDL TG and low HDL levels with lower BMD. In addition, CRI-II and ambulation were identified as predictors of reduced BMD in KD groups, compared to AIP which was the only predictor of reduced BMD in AEDs group. Regrettably, the interplay between dyslipidemia and bone health was not addressed in pediatrics generally and in children with RE particularly yet.

Our results reported growth impairments in patients receiving KD as well as those on AEDs only with significant prevalence of stunting on MAD group and significant wasting in AEDs group. Beyond the impact of AEDs on poor bone health, Prior studies showed that children who used two AEDs at the same time had inadequate growth status as well [43]. While systematic reviews have found inconsistent results on the KD’s effect on growth with some showing a positive impact while others show a negative impact [4, 24].

Etiology of poor growth in children on KD includes energy and protein intake restriction to achieve ketosis, the underlying condition, treatments and acidosis or ketosis. While other studies, specifically those that do not require calorie restriction and provide vitamin and mineral supplementation, do not report such significant impacts on growth [4]. Therefore, constant growth monitoring is necessary for all patients on KD even when used for short periods [56].

Given the propensity of the KD to increase serum lipid levels, lipid profile changes and atherogenic indices should be regularly assessed for their possible risks on both cardiovascular system and bone health. Although cardiac side effects are rare and weren’t reported in our study, should be always suspected and ECGs and echocardiograms are recommended before and during KD therapy. Current practices for supplementation of vitamin D and calcium were not sufficient to prevent BMD reduction so DEXA scan should be performed and correlated with. Larger studies are required to further explore these relationships, and longer term follow up is required to determine the effect of the ketogenic diet on peak bone mass, as well as fracture risk throughout life. Atherogenic indices, particularly AIP should be studied in a well-designed trial to detect its impact on CVD and osteopenia in children. Finally, as RE represents around one third of epileptic children the link between KD and AEDs should be further understood, with frequent monitoring of AEDs long-term consequences.

The main strengths of our study are that to our knowledge the present study is the first to evaluate the long-term impact of KD on cardiac function, ECG changes, carotid artery changes, BMD and growth collectively. Also, we studied two different types of KD with different fat to carbohydrate ratios. For accurate estimation of dyslipidemia risks we used mean levels of lipid profile parameters through 2 years instead of single measurement in addition to atherogenic indices which weren’t previously studied in children with RE and have uprising concerns in predicting atherosclerotic risk than standard lipid profile. Finally, we compared children receiving KD with others receiving multiple AEDs who never received KD before as being the alternative therapy to KD in RE.

### Limitations of the study

The main study limitations include absence of baseline imaging and laboratory parameters for detection if whether abnormalities were present before the start of KD or a consequence after initiation. Also, atherogenic indices in children with RE weren’t studied before so there was difficulty in comparing our results with a similar population. Additionally, all patients received at least 2–3 AEDs before and during KD which may alter the results.

## Conclusion

AIP was elevated in children receiving either KD or AEDs indicating moderate to high atherogenic risk in children with RE in addition was identified as the only predictor for decreased BMD in AEDs group. Also, CRI-II and ambulation were the predictors in KD groups. Therefore, large scale studies are recommended to detect the actual role of atherogenic indices in atherosclerosis and osteoporosis risk in children with RE. No cardiovascular side effects were observed in our study addressing the rarity of these effects in monitored KD, however, follow up remains mandatory. Reduced BMD is an underestimated problem in children with RE and probably has multifactorial etiology indicating the need for further research. Growth impairment was evident in KD and AEDs groups suggesting that KD intake is not the only determinant of abnormal growth in RE children. Yet, KD can be referred to as an effective therapy with side effects therefore patients on prolonged KD should be monitored carefully.

## Supplementary Information

Below is the link to the electronic supplementary material.

Supplementary Material 1

## Data Availability

The datasets used and/or analyzed during the current study are available from the corresponding author on reasonable request.

## Abbreviations

AEDs: Antiepileptic drug
AIP: Atherogenic index of plasma
A C: Atherogenic coefficient
BMD: Bone mineral density
BMI: Body mass index
CVD: Cardiovascular disease risk
CRI-I: Castelli risk index 1
CRI-II: Castelli risk index II
CIMT: Carotid artery intima-media thickness
CAC: Carotid artery compliance
DEXA: Dual- energy X-ray absorptiometry
ECG: Electrocardiogram
HDL-C: High-density lipoprotein cholesterol
ILAE: International League Against Epilepsy
KD: Ketogenic diet
LDL-C: Low density lipoprotein cholesterol
MAD: Modified Atkins diet
QTc: Corrected QT interval
RE: Refractory epilepsy
RDA: Recommended Dietary Allowance
SELENBP1: Human Selenium-binding protein 1
SI: Arterial stiffness
TC: Total cholesterol
TG: Triglycerides
VLDL: Very low-density lipoprotein
