# Supplementary material for: Long-term cardiometabolic and bone health consequences of ketogenic diet in children with refractory epilepsy
Source: Ital J Pediatr. 2025 Aug 22;51:261. doi: 10.1186/s13052-025-02109-1 (PMC12374288; doi:10.1186/s13052-025-02109-1)
Supplement: Supplementary file 1 — Supplementary Material 1 [file 13052_2025_2109_MOESM1_ESM.docx]

Dear Editor

There are no comments from reviewers provided during minor revision.

Thanks

Dr Aya
